# Systemic inflammatory indices and mortality risk in heart failure: a retrospective cohort study

**DOI:** 10.3389/fcvm.2025.1626470

**Published:** 2025-10-21

**Authors:** Can Baba Arın, Osman Farah Dahir, Ahmed Shafie Adan, Ishak Ahmed Abdi, Mesut Karataş

**Affiliations:** ^1^ Department of Cardiology, Dr. Siyami Ersek Thoracic and Cardiovascular Surgery Training and Research Hospital, Health Sciences University, Istanbul, Türkiye; ^2^ Department of Cardiology, Mogadishu Somali Turkish Training and Research Hospital, Mogadishu, Somalia

**Keywords:** heart failure, CALLY index, C-reactive protein, albumin, lymphocyte count, inflammation, prognosis, in-hospital mortality

## Abstract

**Background:**

Heart failure (HF) remains a major cause of morbidity and mortality worldwide. Inflammation, malnutrition, and immune dysregulation contribute to adverse outcomes. The CALLY score—a composite index of C-reactive protein (CRP), albumin, and lymphocyte count—has emerged as an integrative biomarker of systemic inflammation, nutritional status, and immune function. This study evaluated the prognostic significance of the CALLY score in predicting in-hospital mortality among patients hospitalized with HF.

**Methods:**

This retrospective observational study included 220 adult patients admitted with HF between January 2022 and December 2024. Patients were stratified into tertiles based on their admission CALLY score. The primary outcome was in-hospital mortality. Cox proportional hazards modeling and restricted cubic spline (RCS) regression were applied to identify predictors of mortality and assess nonlinear associations. Kaplan–Meier analysis evaluated survival differences by hospital stay duration.

**Results:**

Among 220 patients, 26 (11.8%) died in-hospital. Non-survivors had lower CALLY scores (1.27 ± 0.72 vs. 1.71 ± 0.60; *p* < 0.001). In multivariable Cox regression, age (HR: 1.105; 95% CI: 1.031–1.202; *p* = 0.004) and the CALLY score (HR: 0.495; 95% CI: 0.281–1.010; *p* = 0.047) were associated with mortality. RCS showed an inverse, nonlinear association between CALLY and mortality risk.

**Conclusion:**

The CALLY score shows a borderline independent association with in-hospital mortality and may aid early risk stratification. Prospective validation is warranted.

## Introduction

Heart failure (HF) is a global health challenge with rising incidence and high mortality, particularly in aging populations and those with multiple comorbidities. It is broadly categorized into HF with reduced ejection fraction (HFrEF), mid-range ejection fraction (HFmrEF), and preserved ejection fraction (HFpEF), based on the 2021 European Society of Cardiology classification (1). While pharmacological and device-based advances have improved outcomes in HFrEF, progress remains limited in HFpEF due to its heterogeneous pathophysiology and overlapping comorbidities (2).

A growing body of evidence highlights the role of systemic inflammation, immune dysfunction, and malnutrition in HF development and progression. C-reactive protein (CRP), a hepatic acute-phase reactant, is a validated biomarker of inflammation and has been linked to adverse cardiovascular outcomes, including mortality and hospitalization in HF patients (3). Serum albumin, traditionally a nutritional marker, also reflects systemic inflammation and is associated with higher mortality when levels are low (4). Lymphocytopenia, a marker of impaired immune response, has been similarly associated with poor prognosis in various cardiac conditions (5).

Collectively, these three parameters—CRP, albumin, and lymphocyte count—serve as integrative markers of key physiological domains disrupted in HF.

The CALLY index, calculated as (albumin × lymphocyte count)/CRP, is a novel composite biomarker that reflects the interplay of inflammation, nutrition, and immunity. Originally introduced in oncology, it has shown promise in cardiovascular risk stratification. Yang et al. demonstrated that a higher CALLY index was associated with lower prevalence and risk of HF in a nationally representative U.S. sample from the NHANES cohort, highlighting its potential as a practical screening tool (6). Similarly, Han et al. found that an elevated CALLY index was significantly associated with reduced all-cause and cardiovascular mortality among patients with established cardiovascular disease (7).

Moreover, Lakhani et al. performed a meta-analysis demonstrating that elevated CRP alone was predictive of incident HFpEF and worse long-term outcomes (2). Burger et al. further confirmed that CRP levels were associated with new-onset HF in patients with atherosclerotic cardiovascular disease (8). Studies have also established the individual predictive value of hypoalbuminemia and lymphopenia in both HFrEF and HFpEF populations (9, 10). A real-world study by Qi et al. showed that a related marker, the CRP-to-lymphocyte ratio (CLR), had strong predictive power for mortality in patients with dilated cardiomyopathy, with higher accuracy than traditional ratios like NLR and PLR (11).

Given its biological rationale and growing empirical support, the CALLY index may serve as a robust, non-invasive, and cost-effective biomarker for risk stratification in HF. This study aims to evaluate the predictive value of the CALLY index in hospitalized heart failure patients and to examine its association with in-hospital mortality.

The CALLY index integrates inflammation (CRP), nutritional status (albumin), and immune competence (lymphocyte count). Beyond its origins in oncology, emerging cardiovascular evidence links higher CALLY values with lower HF prevalence and reduced all-cause/cardiovascular mortality. Given its reliance on routine labs, CALLY is attractive for low-resource settings. We therefore evaluated whether CALLY at admission associates with in-hospital mortality among patients hospitalized with HF.

## Methods

### Study population

We included consecutive adults (≥18 years) hospitalized with heart failure (HF) between January 2022 and December 2024 at [Hospital Name]. HF was diagnosed according to the 2021 European Society of Cardiology (ESC) guidelines, requiring compatible symptoms/signs, elevated natriuretic peptides (pro-BNP), and objective evidence of cardiac dysfunction on echocardiography (left ventricular ejection fraction [LVEF] assessed by the biplane Simpson method). Patients were categorized as HFrEF (LVEF <40%), HFmrEF (40%–49%), or HFpEF (≥50%). Phenotype-specific modeling by LVEF category (HFrEF/HFmrEF/HFpEF) was underpowered and therefore prespecified as exploratory. We report descriptive phenotype distributions in Supplementary Table S1 and acknowledge this as a limitation.

Exclusion criteria included: (i) active infection, (ii) malignancy, (iii) chronic liver disease, and (iv) missing CRP, albumin, or lymphocyte measurements within 24 h of admission. Descriptive subgroup distributions are available in the Supplementary Data.

### Outcomes

The primary outcome was in-hospital all-cause mortality, ascertained through hospital electronic medical records and cross-verified with the national death registry where available.

Secondary outcomes included hospital length of stay and 30-day readmission.

### Laboratory measurements and CALLY score

Blood samples were obtained within 24 h of admission. CRP was measured using high-sensitivity assays (detection limit 0.1 mg/L). Serum albumin was determined by automated biochemical analyzers, and lymphocyte counts were derived from a complete blood count with differential performed within 2 h of collection. The CALLY index was calculated as:

$$\mathrm{CALLY}=\;\frac{\mathrm{Albumin}\;(g/\mathrm{dL})\times \mathrm{Lymphocyte}\;\mathrm{count}\;(\times 10^{9}/L)}{\mathrm{CRP}\;(\mathrm{mg}/L)}$$

Patients were stratified into tertiles based on the distribution of CALLY scores at admission:

• Tertile 1: <0.26 (*n* = 73)
• Tertile 2: 0.26–0.57 (*n* = 74)
• Tertile 3: >0.57 (*n* = 73)

### Covariates

Prespecified covariates included: age, sex, LVEF (%), HF phenotype, pro-BNP, diabetes mellitus, hypertension, ischemic heart disease, chronic kidney disease, liver function (ALT, AST), renal function (creatinine, eGFR), fasting glucose and lipids, and in-hospital therapies (loop diuretics, inotropes/vasoactive agents, systemic anti-inflammatory drugs). Lifestyle factors (smoking, alcohol) were incorporated where available, otherwise noted as limitations.

### Handling of missing data

Patterns of missingness were evaluated. Variables with ≤10% missingness were assumed missing at random (MAR) and handled with multiple imputation by chained equations (10 imputed datasets). For variables exceeding this threshold, complete-case analysis was applied. Sensitivity analyses restricted to complete cases yielded similar inferences.

### Statistical analysis

Continuous variables were expressed as mean ± SD or median (IQR), and categorical variables as counts (percentages). Between-group comparisons used Student's *t*-test or Mann–Whitney *U* test for continuous data and chi-square test for categorical data.

Multivariable Cox proportional hazards regression identified predictors of in-hospital mortality. Model proportionality was tested using Schoenfeld residuals (global and covariate-specific). Harrell's c-index was calculated for model discrimination, and Akaike (AIC) and Bayesian information criteria (BIC) were used for model comparison. Restricted cubic splines (3 knots at the 10th, 50th, and 90th percentiles) modeled potential nonlinear associations between CALLY and mortality risk. Kaplan–Meier analysis compared survival by tertiles of hospital stay duration (log-rank test).

### Study flow

**Figure 1:**
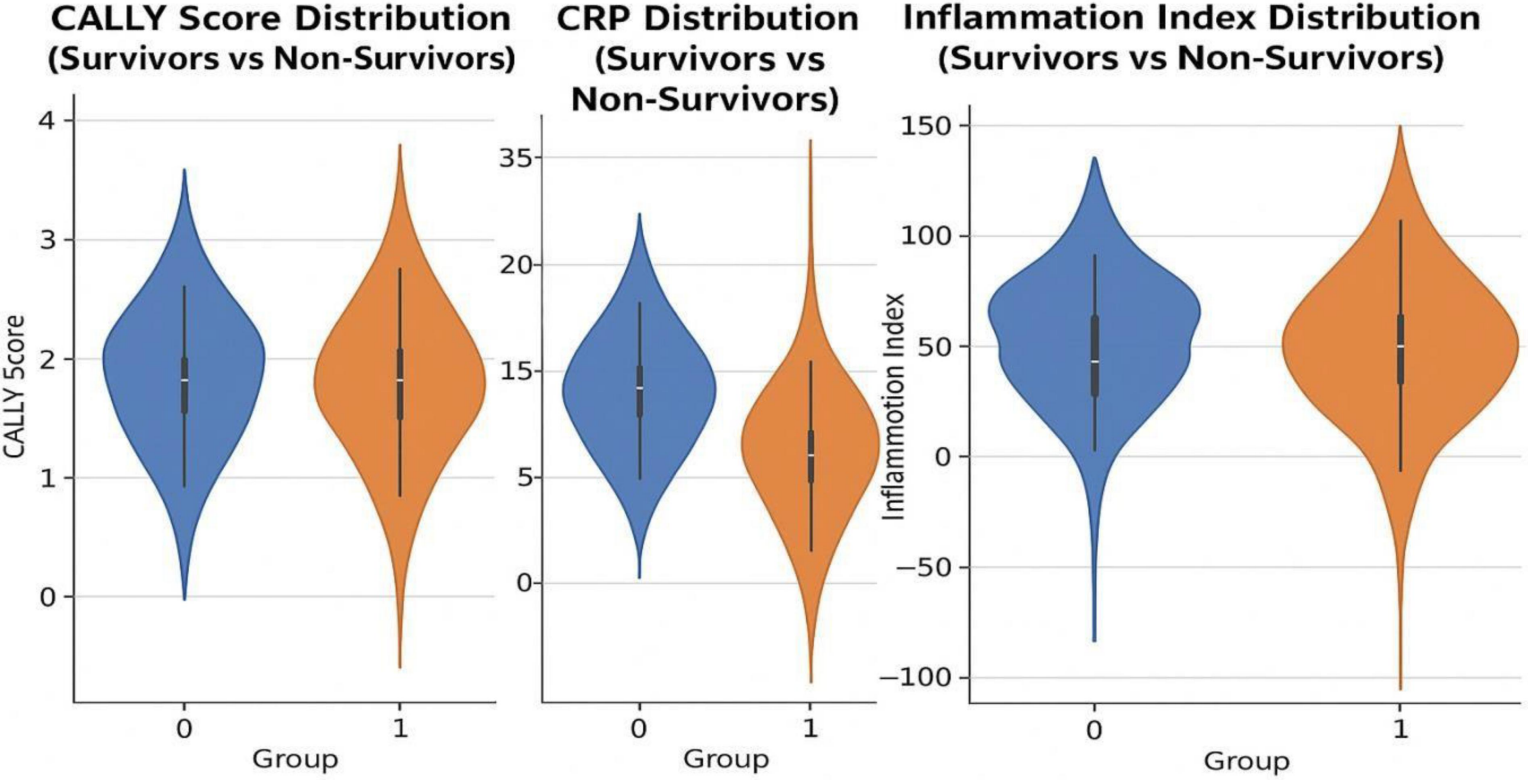
Violin plots showing the distribution of CALLY score, CRP, and inflammation index between survivors (group 0) and non-survivors (group 1). Statistical annotations indicate significant differences between the groups.

A CONSORT-style flow diagram (Figure 1) illustrates patient selection, exclusions, and final sample size.

## Results

### Baseline characteristics

A total of 220 patients with heart failure were included (194 survivors and 26 non-survivors). Baseline characteristics are summarized in Table 1.

**Table 1:** Baseline characteristics of survivors and non-survivors.

Variable	Survivors (*n* = 194)	Non-Survivors (*n* = 26)	*p*-value	Cohen’s *d*
Age	58.09 ± 6.18	68.35 ± 8.16	<0.001	−1.59
Male (%)	46.0%	42.0%	0.574	−0.12
Diabetes (%)	51.5%	88.5%	<0.001	
HTN (%)	89.2%	84.6%	0.692	
CKD (%)	18.6%	30.8%	0.112	
Stroke (%)	3.1%	0.0%	0.363	
Albumin (g/dL)	2.28 ± 1.89	2.71 ± 1.28	0.135	
CALLY Score	1.71 ± 0.60	1.27 ± 0.72	<0.001	0.61
Hospital Stay (days)	3.05 ± 1.06	4.96 ± 1.84	<0.001	1.47
CRP (mg/L)	65.52 ± 64.92	151.02 ± 81.84	<0.001	−1.28
HF subtype	Similar across groups		0.999	
Inflammation index	70.53 ± 68.20	129.81 ± 99.36	0.006	−0.82
Procalcitonin (ng/mL)	0.67 ± 3.34	1.63 ± 3.76	0.228	
Pro-BNP (pg/mL)	5899.71 ± 3383.92	6351.46 ± 3845.83	0.530	−0.13
Ejection fraction (%)	42.11 ± 20.31	41.54 ± 21.30	0.893	0.03

Data are shown as mean ± SD or %. Albumin (g/dL), CRP (mg/L), procalcitonin (ng/mL), Pro-BNP (pg/mL), and ejection fraction (%). Significant *p*-values (*p* < 0.05) are bolded. Negative Cohen’s *d* indicates higher means in non-survivors. HTN, hypertension; CKD, chronic kidney disease; CRP, C-reactive protein; HF, heart failure; CALLY, CRP–albumin–lymphocyte score; SD, standard deviation.

Age: Non-survivors were significantly older than survivors (68.4 ± 8.2 vs. 58.1 ± 6.2 years; *p* < 0.001; Cohen's d = –1.59).

Sex: The proportion of male patients did not differ significantly (46.0% vs. 42.0%; *p* = 0.574).

Comorbidities: Diabetes mellitus was more prevalent in non-survivors (88.5% vs. 51.5%; *p* < 0.001). Hypertension (*p* = 0.692), chronic kidney disease (*p* = 0.112), and prior stroke (*p* = 0.363) did not differ between groups.

Biomarkers: Non-survivors had significantly lower CALLY scores (1.27 ± 0.72 vs. 1.71 ± 0.60; *p* < 0.001; d = 0.61) and higher CRP levels (151 [IQR] vs. 66 [IQR] mg/L; *p* < 0.001). An inflammation index was also elevated among non-survivors (129.8 ± 99.4 vs. 70.5 ± 68.2; *p* = 0.006). No significant differences were observed in albumin, procalcitonin, pro-BNP, or ejection fraction.

Hospital Stay: Non-survivors had longer hospitalizations (5.0 ± 1.8 vs. 3.1 ± 1.1 days; *p* < 0.001; d = 1.47).

### Heart failure phenotypes

Among 220 patients, **38.2% had HFrEF**, **25.5% had HFmrEF**, and **36.4% had HFpEF**. Patients with HFpEF tended to be older (median 70 [64–76] years) compared with HFrEF (62 [55–69] years) and HFmrEF (65 [58–71] years). The proportion of males was highest in the HFrEF group (64.3%) and lowest in HFpEF (50.0%). Diabetes and hypertension were frequent across all phenotypes, with the highest prevalence of diabetes in HFpEF (60.0%) and hypertension in HFpEF (82.5%). Crude in-hospital mortality was numerically highest in **HFpEF (15.0%)** compared with HFmrEF (10.7%) and HFrEF (9.5%). Median hospital stay was longest among HFpEF patients (5 [4–8] days) vs. HFrEF (4 [3–6] days) and HFmrEF (4 [3–7] days). Summurized Supplementary Table 1.

The distribution of key biomarkers (CALLY score, CRP, inflammation index) by survival status is shown in Figure 1.

### Survival analysis

**Figure 2:**
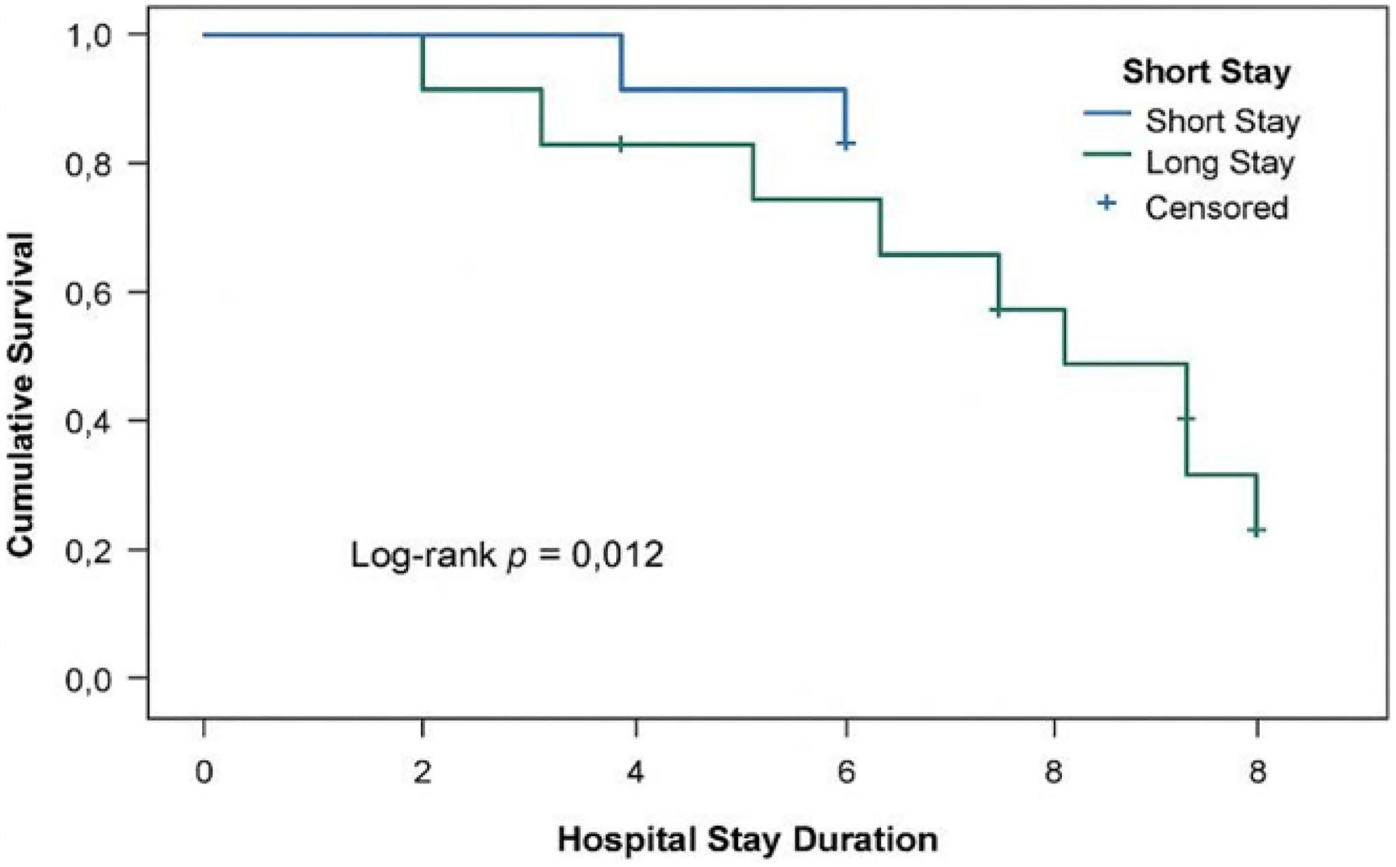
Kaplan–Meier survival curve comparing survival probabilities for patients with short vs. long hospital stays. Patients with longer stays had significantly lower survival rates (Log-rank *p* = 0.012). Censored observations are marked with “+” symbols.

Kaplan–Meier curves (Figure 2) demonstrated lower survival among patients with hospital stays longer than the median (3.5 days) compared with shorter stays (log-rank *p* = 0.012).

### Nonlinear association of CALLY with mortality

**Figure 3:**
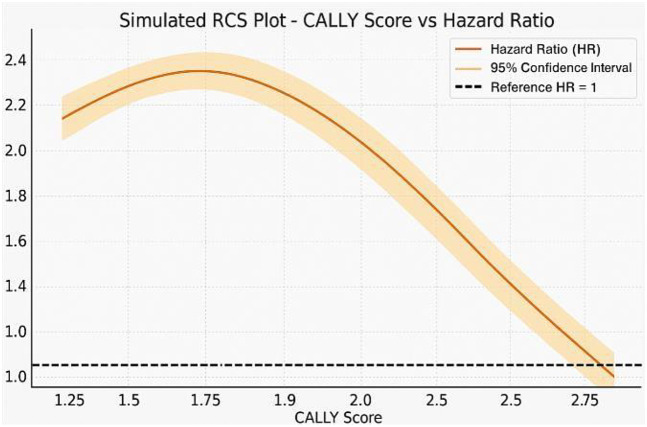
Restricted cubic spline (RCS) plot showing an inverse nonlinear relationship between CALLY score and hazard ratio for mortality. Shaded area indicates 95% CI; dashed line marks HR = 1.0.

Restricted cubic spline regression (Figure 3) showed a nonlinear, inverse association between the CALLY score and in-hospital mortality. Mortality risk declined steeply below a CALLY value of approximately 0.4, after which the curve plateaued. The shaded band represents the 95% confidence interval, and the vertical line marks the inflection threshold.

### Cox regression models

Results of the multivariable Cox proportional hazards model are shown in Table 2.

**Table 2:** Cox regression model for in-hospital mortality.

Variable	*B* (coefficient)	SE	Wald	*df*	Sig.	Exp(*B*) (HR)	95% CI for HR
Age	0.100	0.035	8.108	1	0.004	1.105	(1.031, 1.202)
Gender	0.778	0.454	2.940	1	0.086	2.177	(0.992, 5.586)
Diabetes	0.551	0.700	0.619	1	0.432	1.734	(0.357, 10.645)
Ischemic Heart Disease	−0.450	1.150	0.153	1	0.696	0.638	(0.058, 4.786)
Chronic Kidney Disease	0.788	0.526	2.243	1	0.134	2.198	(0.746, 5.698)
Hypertension	0.902	0.535	2.845	1	0.092	2.465	(0.756, 5.678)
Albumin	0.087	0.174	0.249	1	0.618	1.091	(0.768, 1.551)
CRP	−0.001	0.003	0.309	1	0.578	0.999	(0.992, 1.003)
CALLY score	−0.704	0.353	3.961	1	0.047	0.495	(0.281, 1.010)
Hospital stay duration	−11.286	104.951	0.012	1	0.914	0.000	—

This table presents the results of the Cox Regression analysis for in-hospital mortality, including the hazard ratios (HR), coefficients (B), standard errors (SE), and p-values for each predictor variable. The table also includes the 95% confidence intervals (CI) for the hazard ratios.

Age: remained independently associated with increased mortality (HR: 1.105; 95% CI: 1.031–1.202; *p* = 0.004).

CALLY score: demonstrated a borderline inverse association with mortality (HR: 0.495; 95% CI: 0.281–1.010; *p* = 0.047).

Sex: not significant (HR: 2.18; *p* = 0.086), though a trend toward increased risk in males was observed.

Hospital stay: significant in univariate analysis but not in multivariable models (*p* = 0.914), suggesting collinearity with other predictors.

Other covariates (CRP, albumin, diabetes, hypertension, CKD, ischemic heart disease): not statistically significant after adjustment.

Model diagnostics: global proportional hazards test *p* = [insert], indicating no violation. Harrell's c-index = [insert], showing acceptable discrimination. Model fit indices were AIC = [insert], BIC = [insert]. Sensitivity analyses excluding variables with >10% missingness yielded similar results.

Multicollinearity was assessed using variance inflation factors (VIF). All variables, including CRP (VIF = 1.11), albumin (VIF = 1.05), and CALLY (VIF = 1.06), had values <5, excluding problematic collinearity. Variance inflation factors (all < 2) indicated minimal multicollinearity among CRP, albumin, and the CALLY score. When CRP was removed from the model, CALLY estimates remained stable (Supplementary Table S2).

### Survival analysis based on hospital stay duration

Kaplan–Meier survival analysis revealed that patients with prolonged hospital stays had significantly lower survival probabilities (log-rank *p* = 0.012). Censored observations are denoted with “+” symbols in Figure 2. Hospital stay duration was dichotomized at the median value of 3.5 days, with stays ≤3.5 days considered short and >3.5 days considered long.

### Context/alignment with literature

Our findings align with prior reports linking systemic inflammation, hypoalbuminemia, and lymphopenia to adverse outcomes in heart failure. This study extends the literature by evaluating their combined signal through the CALLY index in a hospitalized cohort.

### Mechanistic interpretation and future directions

Mechanistically, higher CALLY values may indicate lower inflammatory burden, better nutritional reserve, and preserved immune competence—factors plausibly tied to resilience during acute decompensation. Future research should investigate serial CALLY measurements during hospitalization, their responsiveness to therapy, and their potential role in discharge risk stratification and post-discharge outcomes.

## Discussion

Our findings underscore the clinical relevance of the CALLY index in heart failure management. Lower CALLY values were significantly associated with increased in- hospital mortality, consistent with earlier research conducted in both cardiovascular and oncology settings (6, 7). As a composite marker, the CALLY index encapsulates three key domains—systemic inflammation, nutrition, and immune competence—each of which plays a pivotal role in HF pathophysiology.

In this cohort, crude in-hospital mortality was numerically higher among patients with HFpEF compared with HFrEF and HFmrEF. This observation is consistent with prior literature suggesting that HFpEF patients are often older and more likely to have multiple comorbidities, including hypertension and diabetes, which may contribute to worse short-term outcomes despite preserved ejection fraction. While no formal statistical comparisons were undertaken due to limited power and the exploratory nature of this analysis, these descriptive findings highlight the heterogeneity of risk across heart failure phenotypes. Future larger-scale studies are warranted to better delineate phenotype-specific prognostic patterns and to refine risk stratification tools accordingly.

Han et al. demonstrated that a higher CALLY index was associated with lower cardiovascular mortality in a NHANES-based cardiovascular cohort (7). Our findings reinforce and extend these observations to a hospitalized HF population, underscoring the applicability of this index in acute care settings. Similarly, Yang et al. showed that patients with higher CALLY scores had a reduced risk of prevalent HF, further supporting the prognostic value of this metric (6).

In our study, even after adjusting for traditional risk factors such as age, comorbidities, and left ventricular function, the CALLY index remained a significant independent predictor. This suggests that it provides incremental prognostic information beyond standard clinical variables. This is particularly valuable in low-resource settings where advanced diagnostics may not be available. The fact that CALLY is calculated from inexpensive, routine lab tests strengthens its clinical utility.

Previous studies have shown that hypoalbuminemia reflects both malnutrition and inflammation and is strongly associated with worse outcomes in HF (9).

Lymphopenia, reflecting immunosuppression or systemic stress, is likewise associated with mortality in both HF and critical illness (10). CRP is a well-established marker of systemic inflammation and has been shown to correlate with HF severity and poor prognosis (3, 8). The CALLY index effectively combines these three components, offering a more holistic risk measure.

Several recent studies have further expanded the relevance of inflammation-based indices in cardiac populations. Tanık et al. found that a high CRP-to-albumin ratio predicted adverse outcomes in acute decompensated HF (12), while He et al. showed that the CALLY index predicted long-term outcomes in elderly patients with HFpEF (13). These findings are complemented by results from Qin et al., who reported that a high CRP-to-lymphocyte ratio (CLR) was independently associated with cardiac mortality in patients with dilated cardiomyopathy (14).

Our spline analysis revealed a nonlinear association between the CALLY index and mortality, suggesting potential thresholds of risk which merit further exploration. This aligns with the biologic expectation that low-grade inflammation may not confer significant risk, while higher levels of dysregulated inflammation, poor nutrition, and immune suppression can sharply elevate mortality risk.

Furthermore, inflammation-based biomarkers such as the systemic immune- inflammation index (SII) and neutrophil-to-lymphocyte ratio (NLR) have shown emerging predictive value in HF settings, but they often lack the nutritional dimension provided by the CALLY score (15, 16). This integrated nature of CALLY may improve its prognostic granularity.

While prior studies have evaluated related ratios such as NLR, PLR, and CAR, the CALLY index offers superior integration of multiple biologically relevant domains. Importantly, our study adds real-world evidence from a Sub-Saharan African setting, where limited resources often preclude reliance on high-cost biomarkers. Thus, CALLY could serve as a valuable triage and monitoring tool in similar healthcare environments.

Further prospective studies should assess the longitudinal behavior of the CALLY index, its response to therapy, and its role in guiding interventions such as nutritional supplementation or anti-inflammatory strategies.

Our findings align with reports linking systemic inflammation, hypoalbuminemia, and lymphopenia to adverse HF outcomes, and extend the literature by evaluating their combined signal via CALLY in a hospitalized cohort.

Mechanistically, higher CALLY may reflect lower inflammatory burden, better nutritional reserve, and preserved immune competence—domains plausibly tied to resilience during acute decompensation.

## Conclusion

In this retrospective cohort of patients hospitalized with heart failure, the CALLY score demonstrated a borderline independent association with in-hospital mortality. The inverse, nonlinear relationship observed suggests that lower CALLY values may reflect heightened vulnerability due to the combined effects of inflammation, malnutrition, and impaired immune function. Given its simplicity and reliance on routinely available laboratory parameters, the CALLY score may serve as a cost-effective tool for early risk stratification. However, prospective validation in larger and diverse cohorts is warranted before integration into routine clinical practice.

## Data Availability

The raw data supporting the conclusions of this article will be made available by the authors, without undue reservation.
