# Supplementary material for: Systemic inflammatory indices and mortality risk in heart failure: a retrospective cohort study
Source: Front Cardiovasc Med. 2025 Oct 21;12:1626470. doi: 10.3389/fcvm.2025.1626470 (PMC12584026; doi:10.3389/fcvm.2025.1626470)
Supplement: Supplementary file 1 [file Table1.pdf]

Supplementary Table S1. *Distribution of heart failure phenotypes and crude in-hospital outcomes. Patients were categorized by left ventricular ejection fraction (HFrEF <40%, HFmrEF 40–49%, HFpEF ≥50%) according to the 2021 ESC Guidelines. Values are shown as n (%) for categorical variables and median [IQR] for continuous variables. No formal statistical comparisons were performed, as phenotype-specific modeling was prespecified as exploratory and underpowered. These data are provided descriptively to illustrate the distribution of phenotypes and associated crude outcomes.*

Variable	HFrEF (<40%) (n=xx)	HFmrEF (40–49%) (n=xx)	HFpEF (≥50%) (n=xx)	Total (N=220)
**Number of patients, n (%)**	84 (38.2)	56 (25.5)	80 (36.4)	220 (100)
**Age, years [IQR]**	62 [55–69]	65 [58–71]	70 [64–76]	66 [58–72]
**Male sex, n (%)**	54 (64.3)	30 (53.6)	40 (50.0)	124 (56.4)
**Diabetes, n (%)**	38 (45.2)	28 (50.0)	48 (60.0)	114 (51.8)
**Hypertension, n (%)**	60 (71.4)	40 (71.4)	66 (82.5)	166 (75.5)
**In-hospital mortality, n (%)**	8 (9.5)	6 (10.7)	12 (15.0)	26 (11.8)
**ICU admission, n (%)**	14 (16.7)	10 (17.9)	16 (20.0)	40 (18.2)
**Hospital stay, days [IQR]**	4 [3–6]	4 [3–7]	5 [4–8]	4 [3–7]

Notes

- Data are shown as **n (%)** for categorical variables and **median [IQR]** for continuous variables.
- Phenotype-specific statistical testing was not performed (exploratory and underpowered).
- This table is **descriptive** to illustrate patient distribution and crude outcomes across phenotypes.
