# Supplementary material for: Systemic inflammatory indices and mortality risk in heart failure: a retrospective cohort study
Source: Front Cardiovasc Med. 2025 Oct 21;12:1626470. doi: 10.3389/fcvm.2025.1626470 (PMC12584026; doi:10.3389/fcvm.2025.1626470)
Supplement: Supplementary file 2 [file Table2.pdf]

Supplementary Table S2. Multivariable Cox Regression for In-Hospital Mortality: Models With and Without CRP

Variable	HR (95% CI) With CRP	p-value	HR (95% CI) Without CRP	p-value	Variable	HR (95% CI) With CRP	p-value
Age (per year)	1.105 (1.031–1.202)	0.004	1.107 (1.033–1.204)	0.003	Age (per year)	1.105 (1.031–1.202)	0.004
Sex (male)	2.177 (0.992–5.586)	0.086	2.095 (0.964–5.432)	0.091	Sex (male)	2.177 (0.992–5.586)	0.086
Diabetes mellitus	1.734 (0.357–10.645)	0.432	1.682 (0.349–9.997)	0.441	Diabetes mellitus	1.734 (0.357–10.645)	0.432
Hypertension	2.465 (0.756–5.678)	0.092	2.381 (0.741–5.497)	0.098	Hypertension	2.465 (0.756–5.678)	0.092
Chronic kidney disease	2.198 (0.746–5.698)	0.134	2.166 (0.731–5.641)	0.141	Chronic kidney disease	2.198 (0.746–5.698)	0.134
Albumin (g/dL)	1.091 (0.768–1.551)	0.618	1.084 (0.763–1.542)	0.625	Albumin (g/dL)	1.091 (0.768–1.551)	0.618
CRP (mg/L)	0.999 (0.992–1.003)	0.578	–	–	CRP (mg/L)	0.999 (0.992–1.003)	0.578
CALLY score	0.495 (0.281–1.010)	0.047	0.501 (0.284–0.998)	0.046	CALLY score	0.495 (0.281–1.010)	0.047

Hospital stay (days)	0.000 (–)	0.914	0.000 (–)	0.919	Hospital stay (days)	0.000 (–)	0.914

Notes:

- Models adjusted for age, sex, diabetes, hypertension, CKD, albumin, CRP, and CALLY score.
- Variance inflation factors for CRP, albumin, and CALLY all <2, excluding multicollinearity.
- Results were consistent across models, suggesting that CALLY subsumes CRP's contribution.

Multivariable Cox regression models for in-hospital mortality with and without CRP as a covariate. Both models adjusted for age, sex, diabetes, hypertension, chronic kidney disease, albumin, and CALLY score. Variance inflation factors (all <2) indicated minimal multicollinearity among CRP, albumin, and CALLY. Removal of CRP did not materially change the hazard ratio for the CALLY score, suggesting that the CALLY index may subsume CRP's prognostic contribution.
